# Supplementary material for: Monochromatic computed tomography using laboratory-scale setup
Source: Sci Rep. 2023 Jan 7;13:363. doi: 10.1038/s41598-023-27409-6 (PMC9825405; doi:10.1038/s41598-023-27409-6)
Supplement: Supplementary file 1 — Supplementary Information. [file 41598_2023_27409_MOESM1_ESM.pdf]

SUPPLEMENTARY MATERIAL TO ”Monochromatic computed tomography using laboratory-scale setup” (Honkanen et Huotari)

Bad or defunct pixels of the detector were removed from the raw image by replacing them with the median of valid 3 x 3 pixels surrounding each bad pixel. In total 20 bad pixels were identified.

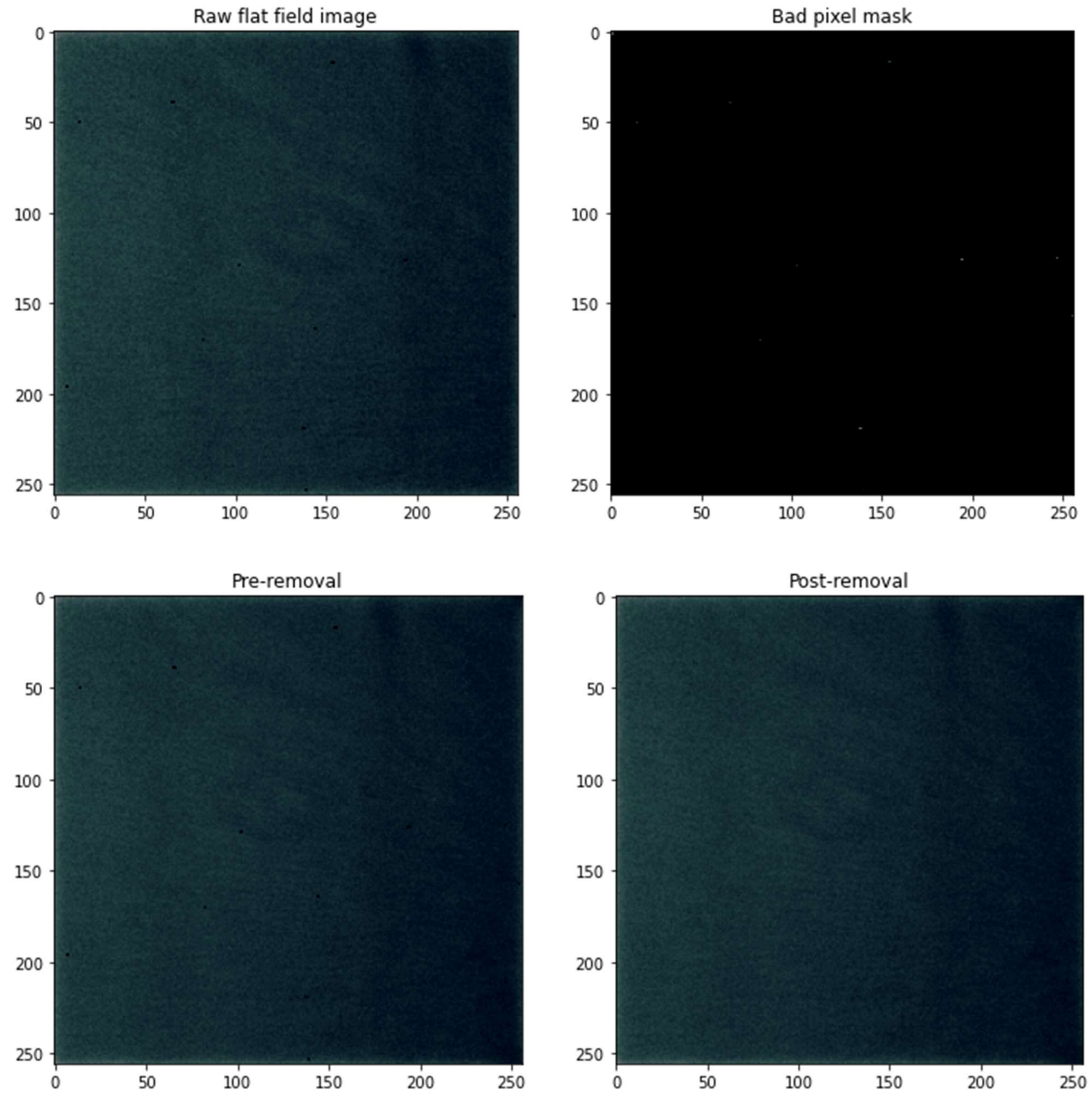

Fig S1: The top row represents the flat field image at 12.645 keV (below all edges) with the dead pixels and the mask idenfying their locations. The bottom row shows the difference in the same flat field image before and after bad pixel removal

Projections are normalized with the flat field image to remove the inhomogeneities in the direct beam.

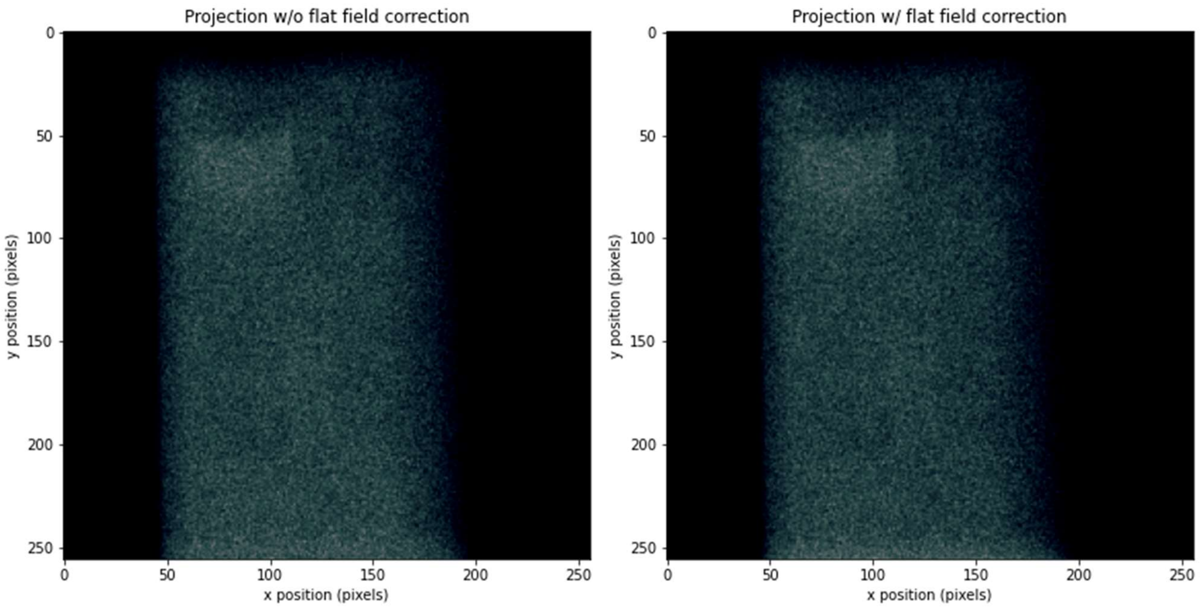

Fig S2: Example projections with an without flat field correction. The left figure is normalized with the mean value of the flat field.

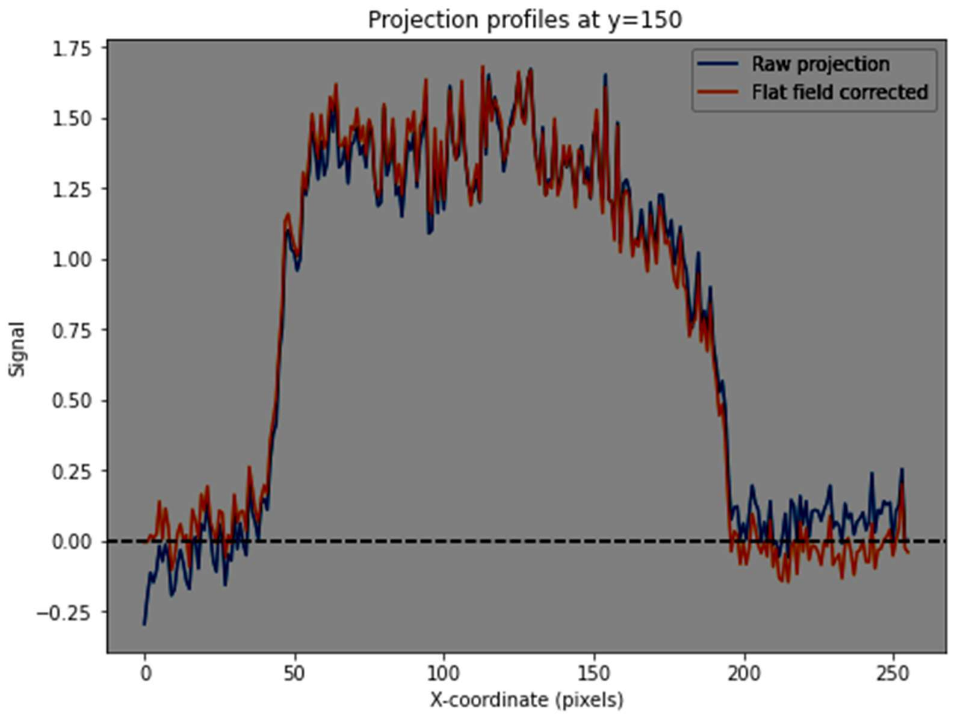

Fig S3: Profiles of the projections in S2. Flat field correction can be seen to level the background at the left and right hand sides of the plot. Dashed horizontal line represents the zero level.

Even after normalization with the flat field, an asymmetric background can be seen in the projections and thus in the sinograms as below:

Fig S4: Example

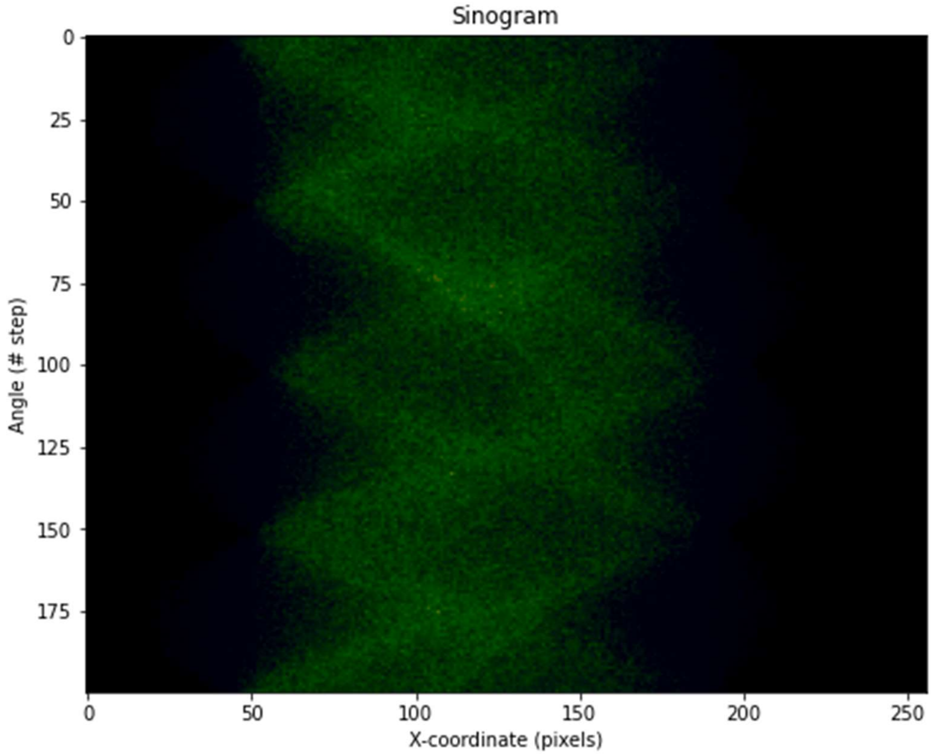

sinogram showing a slight difference in the background levels on left and right hand sides of the image.

Therefore a linear function was fitted to the median (y-direction) background of the slices and removed from the data. The fit and subtracted data is presented below.

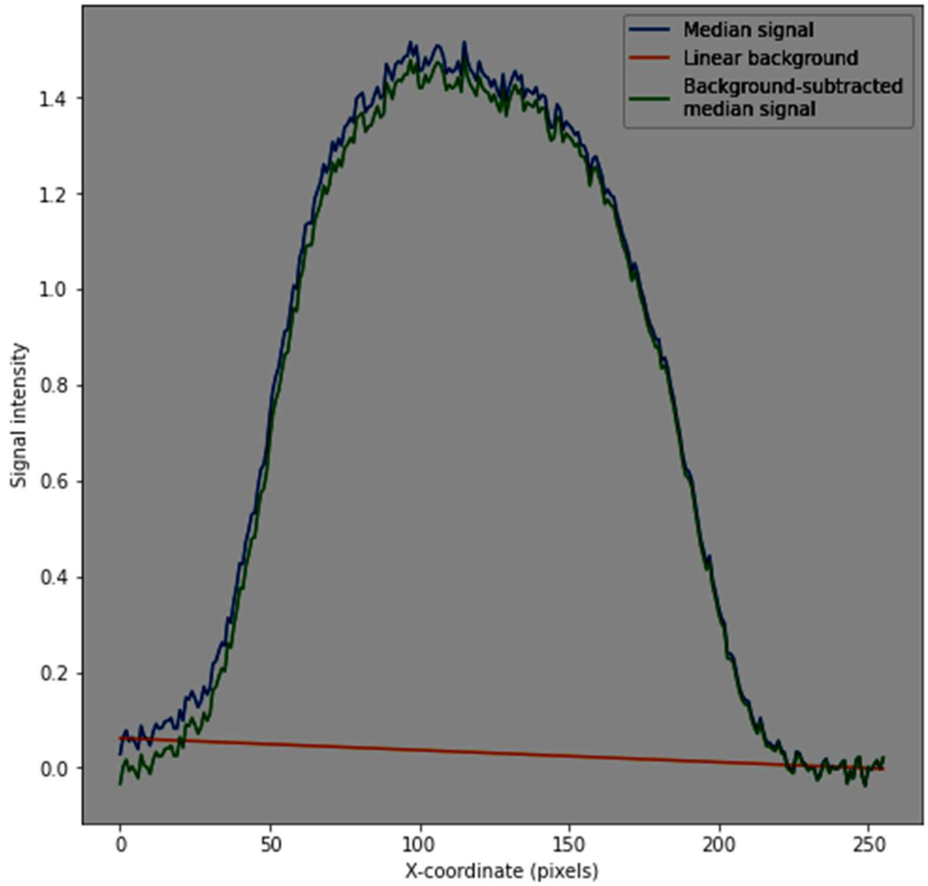

Fig S5: Median signal (y-direction) showing a slight difference in the background levels on left and right hand sides of the image. Linear background is fitted to the median and and it is subtracted from each y-slice.

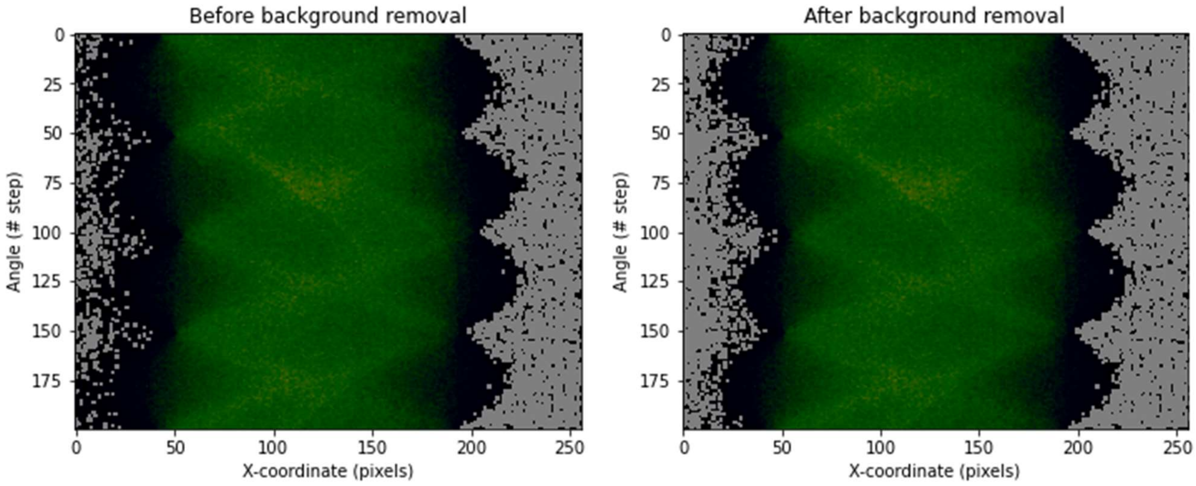

Fig S6: Sinograms before and after the background subtraction. Square root of the sinograms is taken before plotting (white indicates negative values.)

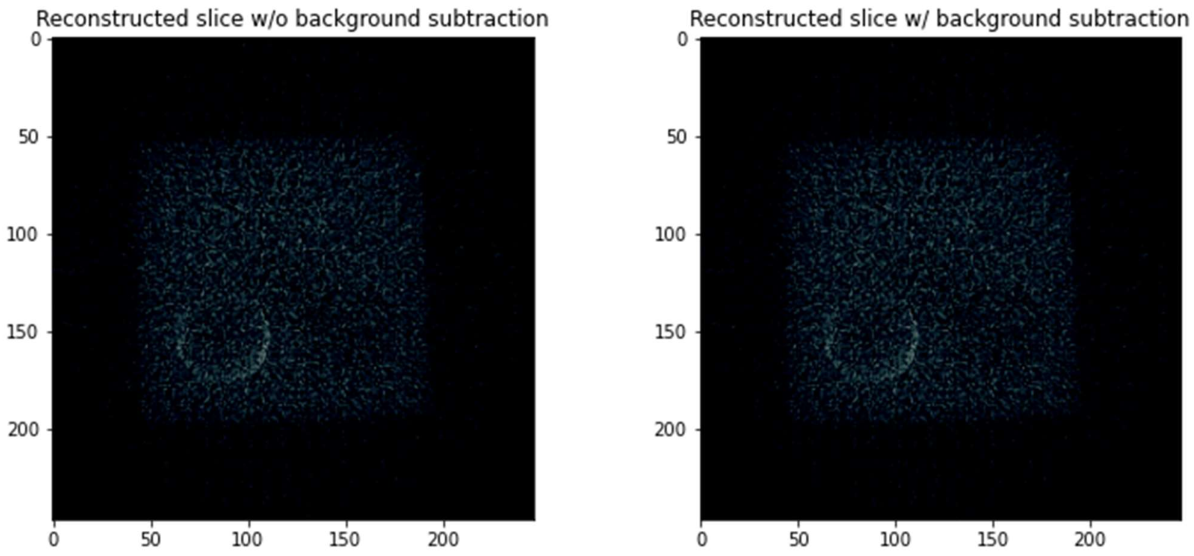

Fig S7: Reconstructions with and without background subtraction. The difference is slight and can not be seen visually

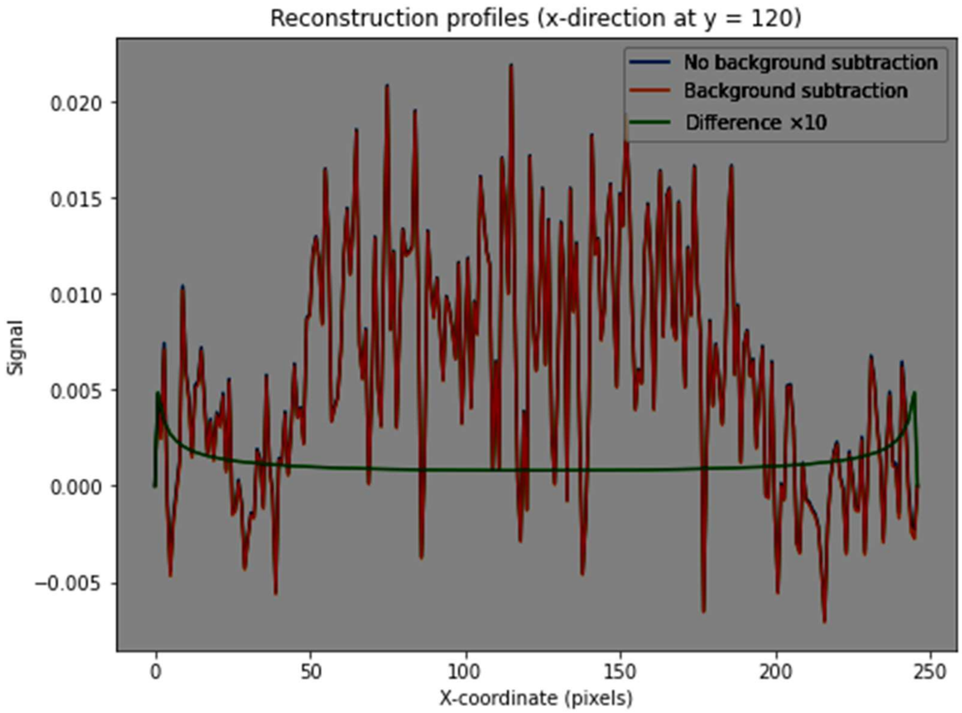

Fig S8: Profiles of the reconstructions in Fig. S7 and their difference scaled by a factor of 10. The background produces a small amount of excess signal which is flat in the center but increases slightly towards the edges of the reconstruction.

The sinogram was separated into 0-180 deg and 180-360 deg subsinograms and the reconstructions were performed separately. Approximately +- 40 % difference was observed at the edges of the

phantom which may be a result of that the beam is not parallel enought for the parallel beam reconstruction.

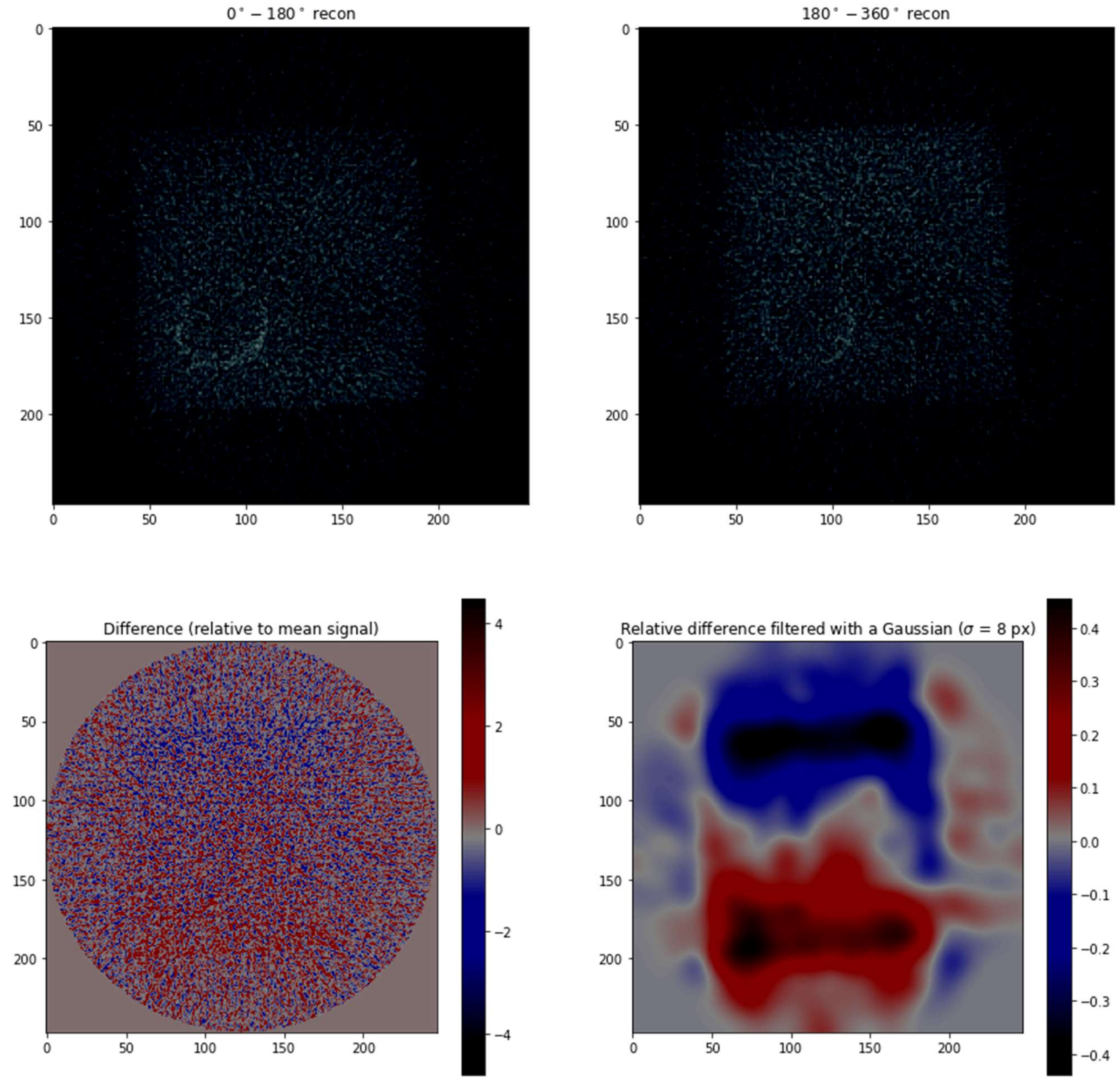

Fig S9: Half reconstructions and their difference. The width of the filter was chosen large enough so that the difference values were converged. The mean of the signal was chosen from the center of the phantom in 130 x 130 px square area.
